# Supplementary material for: A Cre-dependent reporter mouse for quantitative real-time imaging of protein kinase A activity dynamics
Source: Sci Rep. 2024 Feb 6;14:3054. doi: 10.1038/s41598-024-53313-8 (PMC10847463; doi:10.1038/s41598-024-53313-8)
Supplement: Supplementary file 1 — Supplementary Figure S1. [file 41598_2024_53313_MOESM1_ESM.pdf]

A Cre-dependent reporter mouse for quantitative real-time imaging of Protein Kinase A activity dynamics

Elizabeth I. Tilden[1,2], Aditi Maduskar[1], Anna Oldenborg[1], Bernardo L. Sabatini[3], Yao Chen[1,*]

1. Department of Neuroscience, Washington University in St. Louis, St. Louis, MO, United States
2. Ph. D. Program in Neuroscience, Washington University in St. Louis
3. Howard Hughes Medical Institute, Department of Neurobiology, Harvard Medical School, Boston, MA, United States

* Corresponding author. Email: yaochen@wustl.edu

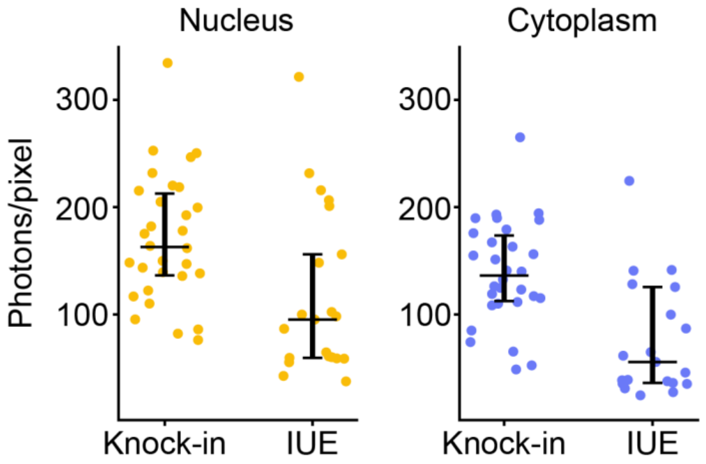

Supplementary Figure S1. Comparison of expression of FLIM-AKAR in *FL-AK* reporter mice vs. in utero electroporation (IUE) delivery of DNA. Quantification of nuclear and cytoplasmic expression from IUE (n = 26 cells from 8 mice) and $Emx1^{IRESCre}$; $FLIM\text{-}AKAR^{flox/flox}$ mice (n = 37 cells from 8 mice). Data are represented as median with 25th and 75th percentiles.
